# Scalp Defect Reconstruction Using the Ying-Yang Double Rotation Flap: A Case Report

**DOI:** 10.7759/cureus.111575

**Published:** 2026-06-26

**Authors:** Diana Laura Páramo Hernández, Miguel Ángel Bretón Gutiérrez

**Affiliations:** 1 General Surgery, Hospital Juárez de México, Mexico City, MEX; 2 Plastic, Aesthetic, and Reconstructive Surgery, Hospital Juárez de México, Mexico City, MEX

**Keywords:** plastic surgery, reconstruction, rotation flap, scalp, ying-yang flap

## Abstract

Scalp reconstruction represents a surgical challenge due to the limited distensibility of tissues and the aesthetic importance of the hair-bearing unit. The ying-yang flap is a double rotation technique designed to optimize defect coverage without resorting to grafts, and the objective of this report is to demonstrate its versatility and efficacy in scalp reconstruction. We present the case of a male patient in his sixties with a lesion secondary to basal cell carcinoma measuring approximately 5 cm in diameter. Following resection, a deep circular defect with exposed pericranium was created. A ying-yang flap technique was performed to achieve a tension-free closure. The postoperative period was uneventful, with preservation of hair density and absence of vascular complications. In conclusion, the ying-yang flap is a versatile and reliable technique that minimizes tissue tension and offers superior aesthetic results in this anatomical region.

## Introduction

The scalp possesses a highly complex anatomical and functional organization, playing a fundamental aesthetic role and acting as a protective barrier for cranial structures. This protective capacity derives from the interaction of its components: the hair follicles; the subcutaneous tissue, composed of adipose tissue; the galea aponeurotica (through which vessels and nerves course); the subgaleal space, consisting of loose connective tissue; and the pericranium or periosteum, a thin, vascularized layer firmly attached to the underlying bone of the cranial vault [1].

From a functional perspective, the scalp protects the skull against direct trauma and external agents. However, its relevance transcends mechanical coverage; the presence of a full-thickness defect exposing deep tissues can compromise tissue viability and promote infectious processes. Without adequate coverage, the risk of developing severe complications such as osteomyelitis, fistulas, or bone perforations with meningeal exposure increases significantly [1].

Surgical planning for the coverage of these defects requires detailed anatomical knowledge as a fundamental pillar. Among the intrinsic factors that pose a considerable challenge for reconstruction are the low elasticity of the galea aponeurotica and the convexity of the cranial vault, elements that significantly restrict the distensibility and availability of tissue for advancement [2]. The primary objectives of reconstruction include achieving stable coverage with richly vascularized tissue, a tension-free closure, and an acceptable aesthetic outcome. To determine the optimal surgical strategy, a comprehensive evaluation must be performed, considering the following: defect characteristics (dimensions, anatomical location, depth, and the integrity of the pericranium), patient factors (systemic comorbidities, history of adjuvant radiotherapy, and the biological quality of the surrounding tissue); and aesthetic considerations (preservation of the hairline, orientation of hair follicles, and regional symmetry). In this context, the choice of technique must be individualized to ensure functional and cosmetic success, ranging from primary closure to more complex procedures, such as rotation flaps [1]. This article highlights the versatility and efficacy of the ying-yang flap as a strategic solution for the optimal closure of full-thickness defects, preserving tissue viability without the occurrence of any postoperative complications.

## Case presentation

Scalp anatomy and vascularization

**Figure 1:**
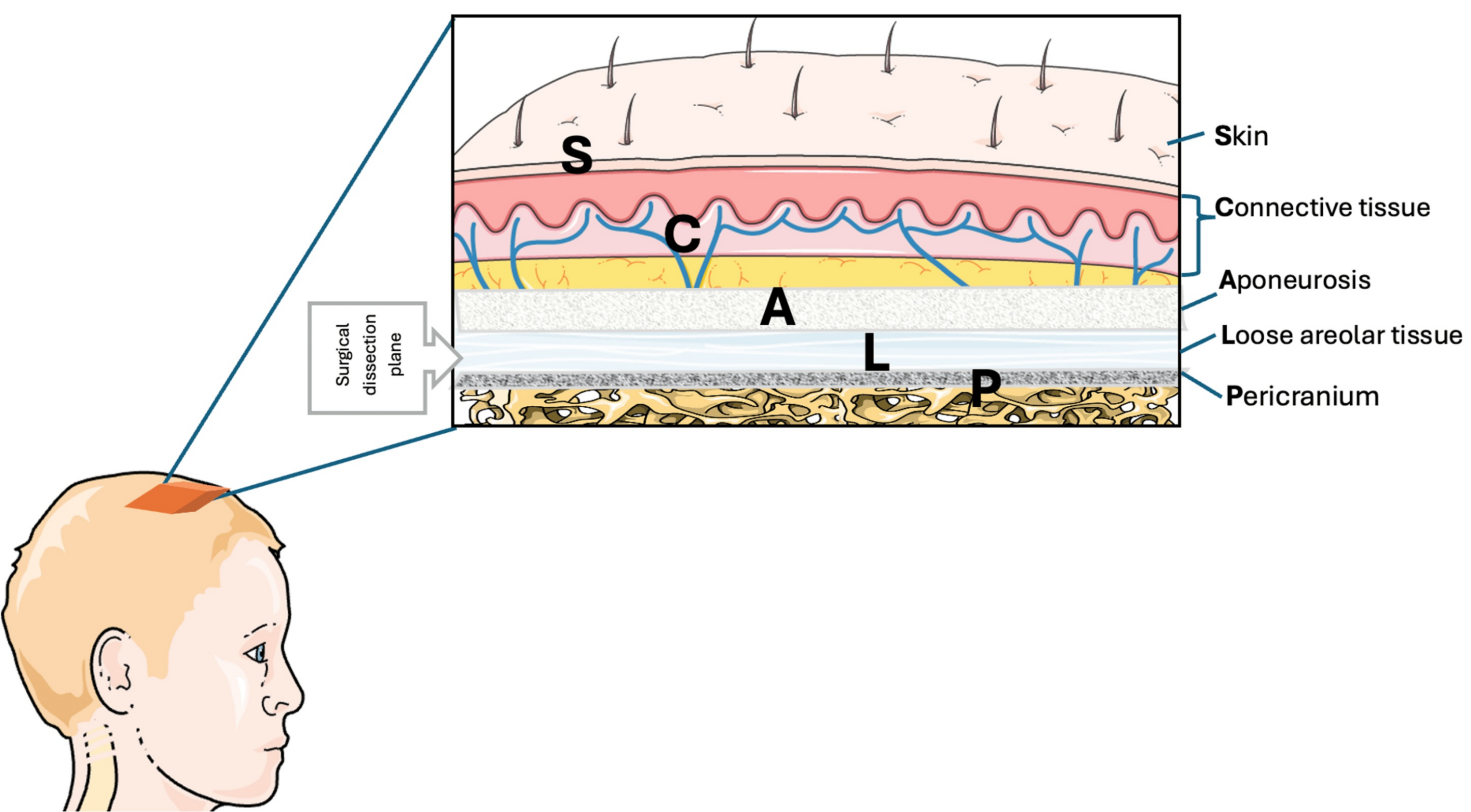
Stratigraphic anatomy of the scalp (SCALP). The loose areolar tissue (L) is highlighted as the ideal avascular dissection plane for flap mobilization. Adapted from image provided by Servier Medical Art (https://smart.servier.com), licensed under the Creative Commons Attribution 4.0 International License (CC BY 4.0; https://creativecommons.org/licenses/by/4.0/).

From a histological and functional perspective, the scalp is organized into five well-defined anatomical layers, commonly synthesized using the SCALP mnemonic: S, skin, epidermis and dense dermis, with a high concentration of hair follicles; C, connective tissue, a dense and richly vascularized subcutaneous layer; A, aponeurosis/galea aponeurotica, a resistant tendinous layer connecting the occipital and frontal muscle bellies; L, loose areolar tissue, an avascular gliding plane that allows the mobility of the scalp over the skull; and P, pericranium, the external periosteum of the cranial vault (Figure 1) [3].

Surgical dissection for the creation of rotation flaps, such as the ying-yang flap, is preferably performed through the loose areolar tissue, utilizing this dissection plane to preserve the integrity of the vessels coursing superficial to the galea.

The blood supply to the scalp is bilateral and highly redundant, allowing for the survival of flaps based on both axial and random flow patterns. This arterial supply is distributed into four main vascular territories. They are as follows: (1) Anterior territory: supplied by the supratrochlear and supraorbital arteries, terminal branches of the ophthalmic artery (derived from the internal carotid artery); (2) Lateral territory: dependent on the superficial temporal artery, a branch of the external carotid artery; (3) Posterior territory: supplied by the occipital artery; (4) Posterolateral territory: supplied by the posterior auricular artery.

**Figure 2:**
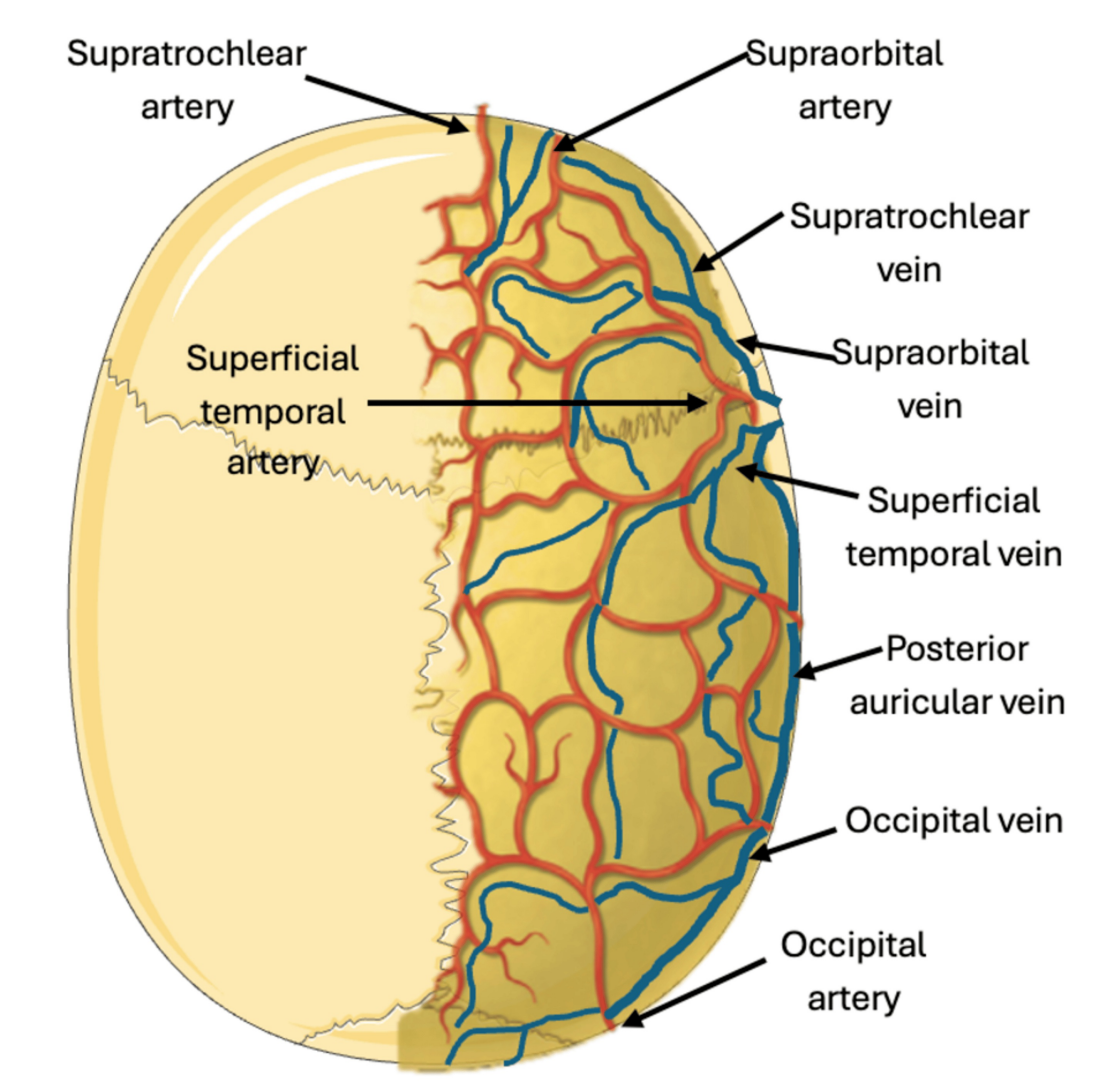
Vascular map of the scalp; Distribution of the arterial and venous territories, highlighting the anastomotic network that ensures the viability of rotation flaps. Adapted from image provided by Servier Medical Art (https://smart.servier.com),  licensed under the Creative Commons Attribution 4.0 International License (CC BY 4.0; https://creativecommons.org/licenses/by/4.0/).

Venous drainage parallels the arterial distribution and shares the nomenclature of the feeding vessels (Figure 2). It is relevant to note that the presence of emissary veins in these planes establishes direct communication with the dural venous sinuses, underscoring the clinical importance of intact cutaneous coverage to prevent the spread of infectious processes into the intracranial space [4].

Surgical design considerations and coverage planning

The planning of scalp defect reconstruction is founded upon the precise determination of the lesion's dimensions and location, the evaluation of the surrounding hair density, and the identification of available vascular pedicles. Additionally, it is necessary to identify factors that may compromise the microvasculature, primarily a history of radiotherapy or peripheral vascular diseases [5].

In cases of oncological etiology, it is a mandatory precept to confirm negative surgical margins via histopathological examination before proceeding with any reconstructive approach.

The selection of the optimal reconstructive technique is based on a rigorous categorization of the defect. As detailed in Table 1, surgical management varies significantly depending on the compromised surface area and regional elasticity, establishing local rotation flaps as the gold standard for defects of moderate extent [1, 6].

**Table 1:** Stratification algorithm for scalp reconstruction based on defect size.

Defect Category	Area (cm²)	Suggested Surgical Technique	Key Considerations
Small	< 5 cm^2^	Primary closure	Evaluation of biparietal laxity and marginal tension.
Medium	5 – 40 cm^2^	Local rotation (e.g., ying-yang) or advancement flaps	Mandatory subgaleal dissection to optimize distensibility.
Large	40 – 90 cm^2^	Broad-based rotation (e.g., O-Z) or multiple flaps	Possible requirement for relaxing incisions (galeotomies).
Extensive	> 90 cm^2^	Free flaps or tissue expansion	Consider microsurgery or multi-stage procedures.

Given the limited elasticity of the scalp, a consequence of the structural rigidity of the galea aponeurotica, flaps must be designed with a ratio of 2:1 or up to 3:1 relative to the diameter of the primary defect. Dissection must be carefully executed within the subgaleal plane (loose areolar tissue), which ensures the preservation of the pericranium and protects the supra-aponeurotic vascular network, allowing for extensive and safe tissue mobilization with minimal donor site morbidity [7].

The double rotation flap (ying-yang)

When the defect diameter and the limited distensibility of the surrounding tissue preclude primary closure, the double rotation flap emerges as a versatile reconstructive strategy. This technique allows for the redistribution of tension forces in two opposite directions, maximizing the utilization of available tissue and minimizing the distortion of adjacent aesthetic units [8].

**Figure 3:**
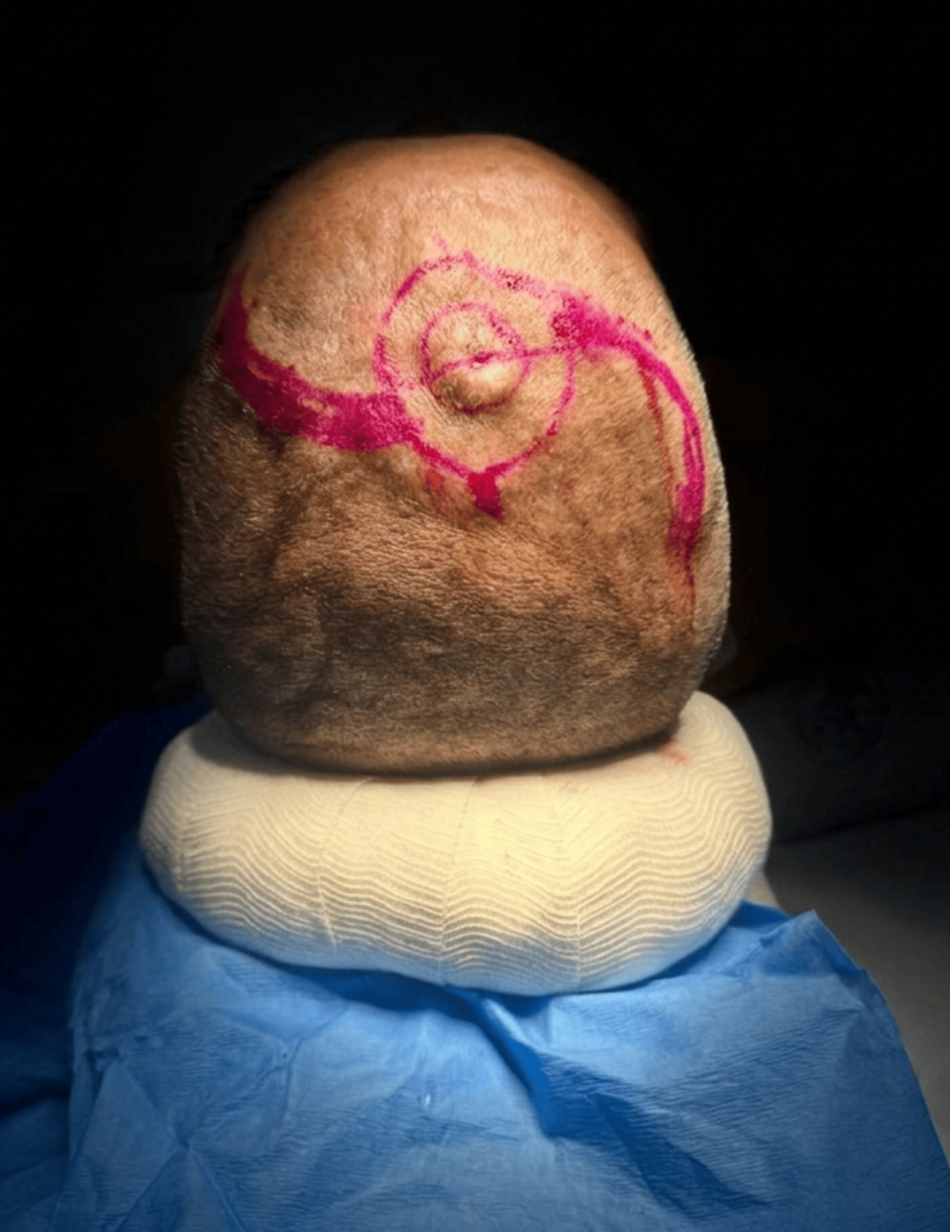
Preoperative planning Surgical marking of the ying-yang double rotation design for a circular defect measuring 5 cm in diameter on the vertex.

We present the case of a male patient in his sixth decade of life, who presented with a lesion consistent with basal cell carcinoma. The lesion was located on the vertex and had a diameter of approximately 5 cm. Following the confirmation of negative margins, reconstruction was planned using a ying-yang type flap (Figure 3).

**Figure 4:**
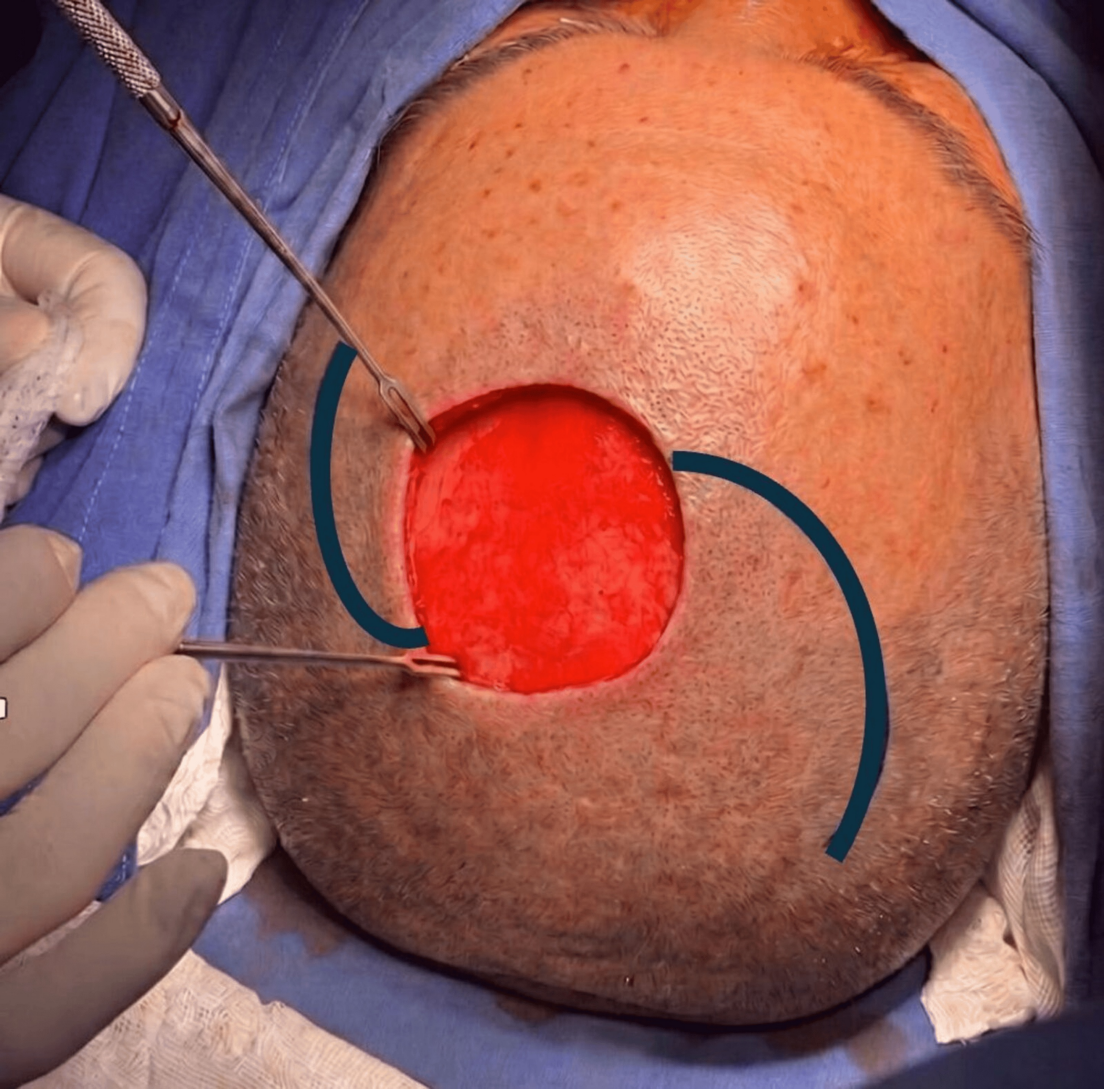
Intraoperative view A 20 cm² circular defect following oncological resection with clear margins. Exposure of the pericranium is observed.

**Figure 5:**
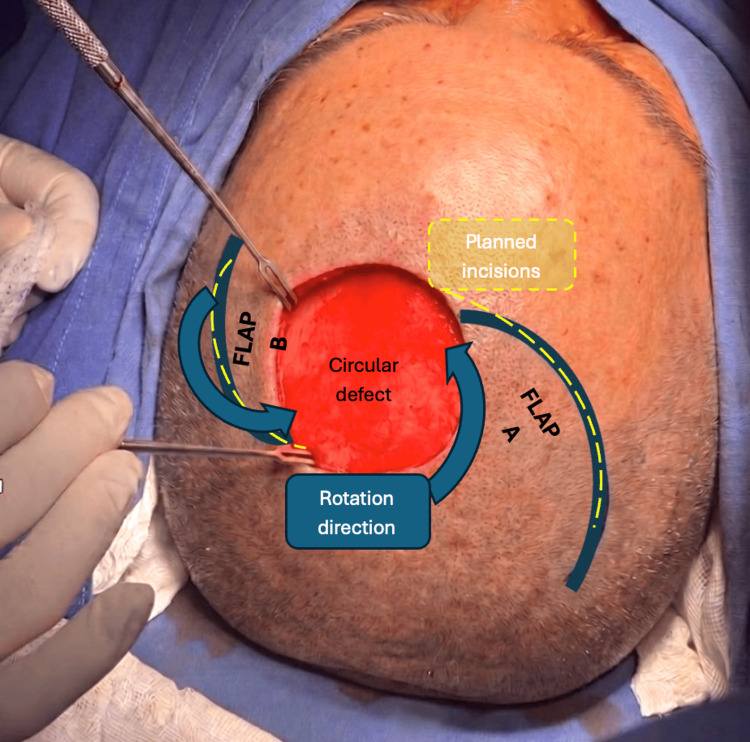
Central circular defect and preoperative markings with yellow dashed lines. The blue arrows indicate the planned direction of rotation for Flap A and Flap B.

Following complete resection of the lesion, a circular defect of approximately 20 cm² resulted (Figure 4). To cover the defect, a double rotation flap was designed. The design consisted of outlining two curved, opposing rotation arcs originating from the margins of the defect, each intended to cover 50% of the wound. This design ensures a symmetrical distribution of mechanical tension, which is a critical factor in an area of low elasticity such as the vertex (Figure 5).

**Figure 6:**
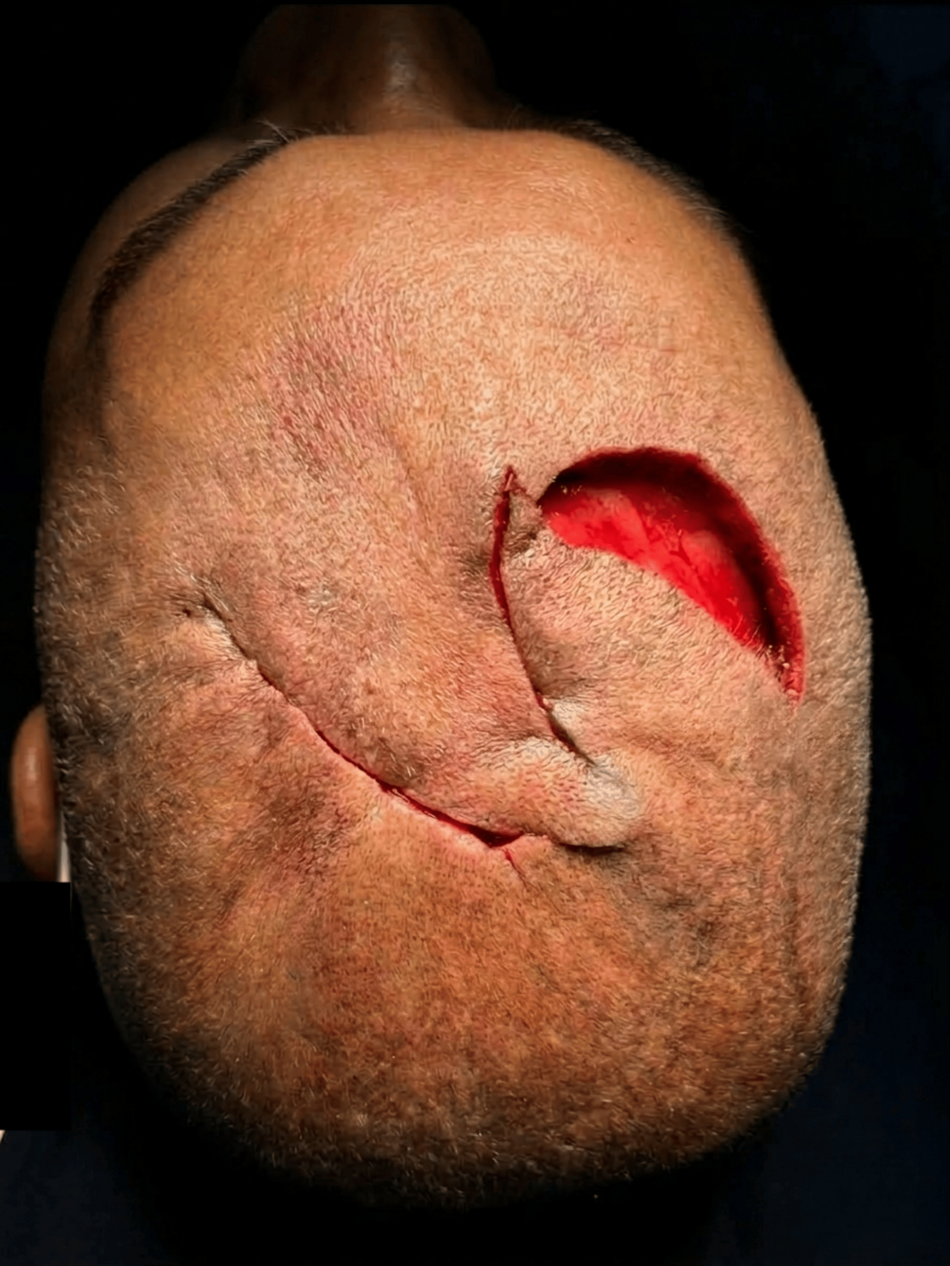
Rotation and approximation; mobilization of both flap arms in opposite directions toward the center of the defect, demonstrating a tension-free closure.

Incisions were made following the pre-established design, deepening the dissection down to the subgaleal plane (loose areolar space). Extensive undermining of both flaps was performed to ensure resistance-free rotation. Subsequently, the advancement and concentric rotation of both arms of the ying-yang design were executed, allowing for central convergence over the defect (Figure 6).

**Figure 7:**
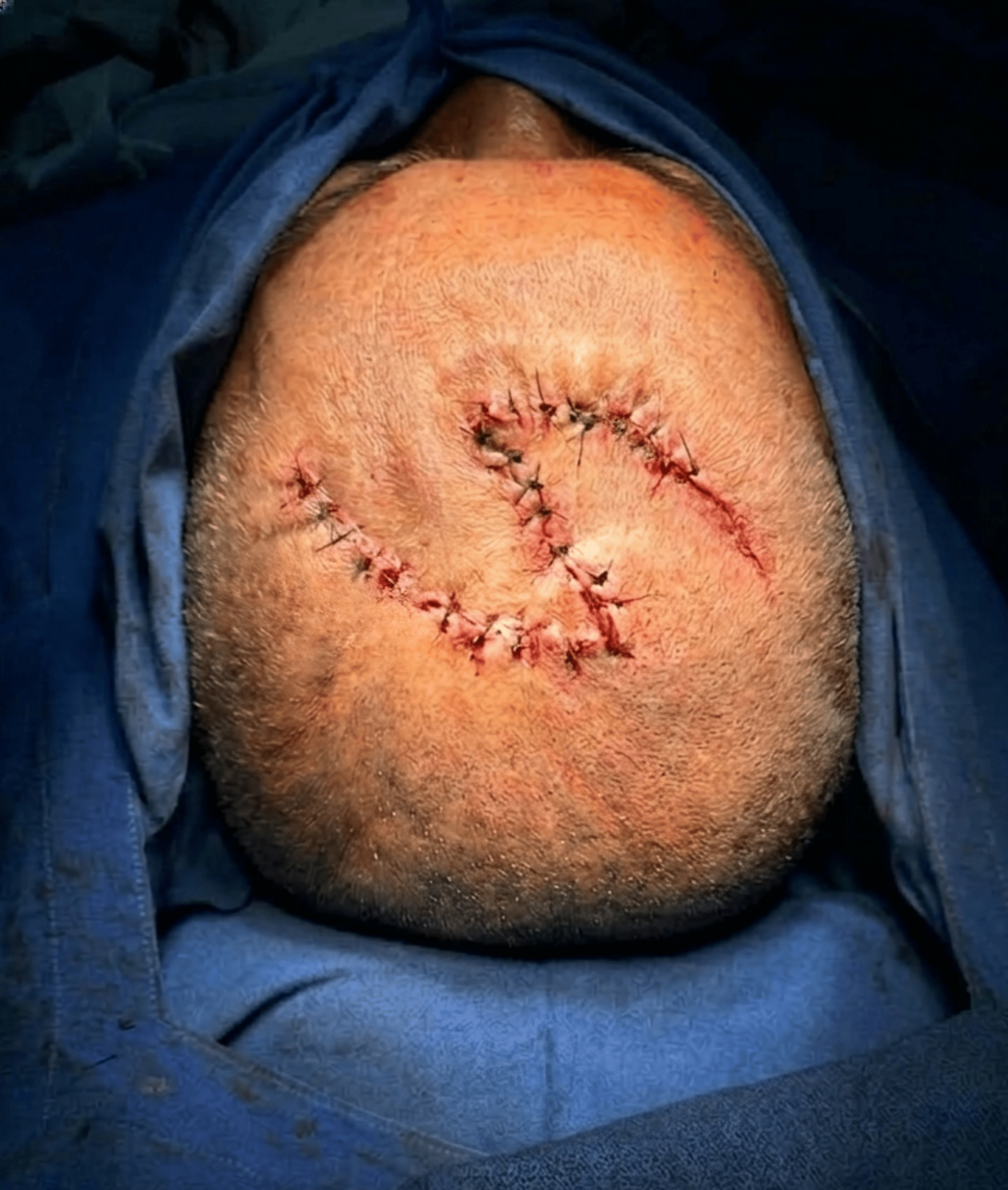
Immediate postoperative view Final closure with monofilament sutures. Preservation of hair follicle orientation and the absence of dog-ear deformities are observed.

To ensure the stability of the reconstruction and minimize the risk of dehiscence, the galea aponeurotica was approximated using inverted absorbable sutures, thereby reducing tension on the dermal margins. Finally, skin closure was completed with simple interrupted non-absorbable monofilament sutures, achieving adequate wound edge eversion and stable coverage (Figure 7).

The surgical procedure was completed in 120 minutes, with an estimated blood loss of 50 cc. A Jackson-Pratt closed-suction drain was placed to prevent the formation of subgaleal seromas or hematomas.

The patient had an uneventful postoperative course. No ischemic complications, such as epidermolysis or distal necrosis, were observed, nor was there any evidence of wound dehiscence. The flap provided stable coverage and preserved the orientation of the hair follicles. During outpatient follow-up, the drain and sutures were removed, confirming an optimal aesthetic result with minimal distortion of follicular growth.

## Discussion

The reconstruction of scalp defects represents a challenge, particularly in elderly patients, due to the progressive decrease in tissue elasticity and the fragility of the microvasculature. In this case, the location of the lesion on the vertex posed an additional challenge, given that the scalp in this region possesses an especially rigid galea aponeurotica compared to other areas of the cranial vault.

The use of the ying-yang type double rotation flap overcame the limitation of local elasticity. Unlike a single rotation flap, which requires a significantly more extensive arc to cover the same defect, the ying-yang technique proportionally distributes the closure tension between two opposing vectors. This configuration drastically reduces cutaneous redundancy (dog-ear deformities), a frequent complication in this surgery [3].

A critical aspect in the resolution of this case was the aesthetic and functional preservation. In male patients, maintaining the orientation of the hair follicles and the integrity of the hairline is fundamental for the cosmetic outcome. The literature suggests that for medium to large defects, closure using a split-thickness or full-thickness skin graft is a viable option; however, this alternative results in a permanent area of alopecia and a contour defect that is often unacceptable to the patient. The ying-yang flap, by utilizing adjacent autologous tissue, ensures coverage with texture, color, and hair density characteristics identical to those of the recipient area [4].

From a vascular perspective, the viability of this flap is supported by the integrity of the anastomotic arcades (branches of the superficial temporal and occipital arteries). By performing the dissection in the subgaleal plane (loose areolar space), it is ensured that the subdermal plexus and axial vessels remain intact within the body of the flap. This is particularly relevant in the elderly population, where distal perfusion may be compromised by subclinical arteriosclerotic changes.

Regarding size limits, the ying-yang flap readily covers defects larger than 5 cm², being optimal for medium defects (5-40 cm²). However, intraoperative assessment of marginal tension is crucial. If the closure is excessively tight, the subgaleal dissection must be extended and galeotomies performed to maximize tissue distensibility and prevent complications such as distal ischemia or local alopecia.

Finally, the ying-yang technique proved to be an effective strategy for achieving a "tension-free closure." The control of mechanical tension is the most important preventive factor against wound dehiscence and postoperative cicatricial alopecia. In this patient, the redistribution of forces allowed for healing by primary intention without compromising cutaneous perfusion, validating the versatility of this design for circular defects where a single flap would be insufficient.

## Conclusions

Scalp reconstruction following the resection of oncological lesions requires a critical balance between surgical radicality and the preservation of functional and aesthetic integrity. The presented clinical case demonstrates that the ying-yang double rotation flap is an exceptionally versatile tool for resolving intermediate-sized circular defects in regions with limited distensibility, such as the vertex. By redistributing tension forces through two opposing rotation vectors, this technique allows for the closure of defects that would otherwise require skin grafts or tissue expanders. Furthermore, by preserving the loose areolar plane and leveraging the scalp's rich vascular anastomotic network, it ensures stable coverage with minimal donor site morbidity and offers a superior cosmetic outcome by maintaining follicular density. Ultimately, the ying-yang flap should be considered a preferential option within the scalp reconstructive algorithm, especially in patients for whom vascular viability and rapid functional recovery are fundamental clinical priorities.
